# Evaluation of a three-phase implementation program in enhancing e-mental health adoption within Indigenous primary healthcare organisations

**DOI:** 10.1186/s12913-020-05431-y

**Published:** 2020-06-23

**Authors:** Buaphrao Raphiphatthana, Michelle Sweet, Stefanie Puszka, Kylie Dingwall, Tricia Nagel

**Affiliations:** Wellbeing and Preventable Chronic Diseases Division, Menzies School of Health Research, Charles Darwin University, PO Box 41096, Casuarina, NT 0811 Australia

**Keywords:** E-mental health, Implementation science, *i*-PARIHS framework

## Abstract

**Background:**

A three-phase implementation program was carried out to support Indigenous primary healthcare organisations in Australia to integrate e-mental health approaches into the day-to-day practice. The present study aimed to evaluate the process and the effectiveness of the program.

**Methods:**

A concurrent triangulation design was employed to collect and compare quantitative and qualitative data from organisations that participated in the implementation program (case studies) to those that participated in training only (non-case studies). Quantitative methods, i.e., t-tests and descriptive statistics, were used to measure outcomes relating to the frequency of e-mental health usage and levels of organisational readiness. Qualitative data were analysed separately, using theoretical thematic analysis, to gain an in depth understanding of the implementation process. The findings were integrated and interpreted within the implementation science literature.

**Results:**

The case studies evidenced greater use of e-mental health approaches than the non-case studies. They also demonstrated increased organisational readiness over the course of the implementation program. The program helped organisations to work and improve on essential aspects within the organisation so that they better supported e-mental health adoption. The key areas addressed were Information Technology resources and infrastructure, leadership and support, policy and protocols around e-mental health utilisation and its integration into practice.

**Conclusions:**

By addressing and improving essential aspects relating to e-mental health implementation, the program helped organisations to increase organisational readiness and enhance uptake of e-mental health approaches.

## Background

e-Mental health (eMH) approaches have exponentially gained popularity in the past decade. The term eMH describes the use of information and communication technology to support and improve mental health conditions and mental health care [1]. Some examples of eMH approaches are online therapy, video/phone counselling, apps (on smartphones or tablets), and websites giving information and promoting mental health and wellbeing. The approach has garnered much attention due to its many benefits, e.g., flexibility, accessibility, cost-efficiency and interactivity [2, 3]. It also provides a promising strategy to overcome traditional barriers such as geographical distance and enable vulnerable individuals to access mental health support.

In the literature, growing evidence demonstrates the effectiveness of eMH approaches in addressing mental health concerns. Many studies have shown that eMH is effective in reducing symptoms associated with a range of mental illnesses, e.g., depression, anxiety, and substance misuse [4–6] in non-Indigneous populations. Fewer studies have been conducted with Indigenous populations; however a recent literature review of international studies suggests usefulness of eMH approaches for Indigenous populations, globally [7]. Similarly, findings within Australia suggests that eMH approaches are perceived to be acceptable, findings within Australia suggests that eMH approaches are perceived.

to be acceptable, feasible, and appropriate for Aboriginal and Torres Strait Islander people (the term Indigenous Australians is used hereafter; with acknowledgement and recognition.

of the diversity of Aboriginal and Torres Strait Islander languages and traditions), by both community members and service providers [8–11].

Recognising the potential of eMH approaches, the Australian government released the National e-Mental Health Strategy initiative in 2012, aiming to increase awareness and utilisation of eMH approaches by all Australians [12]. A component of the initiative is the e-Mental Health training and support (eMHPrac) project which is a joint venture involving multiple Australian institutes aiming to train and promote eMH resources to service providers [13]. As an eMHPrac partner, Menzies School of Health Research (Menzies), in collaboration with University Centre for Rural Health (UCRH) and Queensland University of Technology (QUT), provides eMH training and support to service providers specifically working with Indigenous Australians.

Since October 2013, Menzies has been providing eMH training workshops to organisations working with Indigenous Australians and offers implementation support to all trainees for up to 12 months post-training. Organisations trained include Aboriginal community controlled health services, government community services, primary healthcare network, and non-government community services such as alcohol and other drugs rehabilitation services, and family and carer support services. The training includes e-mental health topics such as confidentiality, quality, credibility, and available eMH resources in Australia. It also provides comprehensive training in the use of the Stay Strong app. The Stay Strong app is a clinician-guided app which uses strengths-based approaches that are culturally responsive to Indigenous Australians’ conceptualisation of wellbeing. The app has been shown to be appropriate, feasible, and acceptable for Indigenous Australians and has received much enthusiasm from both community members and service providers [8–10].

Despite the training effort, a recent report of interviews with trainees revealed that follow-up support alone is not sufficient to ensure succesful uptake. As reported in Raphiphatthana et al. the majority of the trainees indicated that organisational readiness is essential to eMH implementation [14]. Similar to other findings within the field, lack of information technology (IT) resources as well as insufficient policy and procedures to support eMH use were found to impede eMH adoption [15]. Consequently, Raphiphatthana et al. proposed a three-phase implementation program to address organisational readiness for eMH implementation which involved pre-training consultations, training, and follow-up support [14].

The present study aimed to evaluate the effectiveness of the three-phase implementation program in enhancing e-mental health adoption within Indigenous primary healthcare organisations. Organisations which requested e-mental health training were offered the option of an accompanying comprehensive implementation support program or a less intensive version focused primarily on the delivery of training. Data were collected from organisations which enrolled in the implementation program in 2016, 2017 and 2018 (case studies) and those that did not (non-case studies). A detailed description of the differences between the case and non-case studies is outlined below. Underlined by a pragmatic approach, both qualitative and quantitative data were collected and used to evaluate the effectiveness of the implementation program. Particularly, quantitative data were used to answer the following research questions: 1) do case studies evidence greater e-mental health usage than non-case studies? and 2) do case studies evidence improved organisational readiness as they progress through the implementation program? The qualitative data provided further insight into how the implementation program helped to enhance organisational readiness for eMH implementation.

## Methods

### The training workshops and follow-up support

Through the eMHPrac project with partners from UCRH and QUT, Menzies has been conducting face-to-face eMH training workshops for health professionals working with Indigenous Australians at a primary care level, since October 2013. The training workshops are adapted from the Australian Integrated Mental Health Initiative’s (AIMhi) training programme ‘Yarning about Indigenous Mental Health’ which embodies a strength-based approach and incorporates cross-cultural conceptualization of mental health [16]. The programme was further refined through collaborative consultation through the eMHPrac project with partners from the Queensland University of Technology and the University Centre for Rural Health North Coast. The workshops aim to raise awareness and develop skill and confidence in utilisation and referral of e-mental health resources that are responsive to Indigenous cultures.

In addition to eMH training workshops, Menzies also offers a Train the Trainer (TtT) workshop. This workshop is designed to help trainees develop essential skills and knowledge to independently conduct eMH workshops and provide supervision to other staff within their organisation. The purpose of Train the Trainer is to help organisations build the capacity to integrate and maintain using eMH approaches as part of the working culture long-term without having to rely on external training and support. More details regarding the TtT workshop are documented in the facilitators’ guide and learning materials [17].

Post-training, all trainees were offered 12-month follow-up support. Support was provided in the form of emails, phone calls, and face-to-face sessions in order to check progress in implementing e-mental health approaches, to help troubleshoot issues and to further develop skill and confidence in eMH useage. This follow-up support had two purposes: 1) To provide support to staff from organisations who did not have trainers and thus required our support to implement eMH resources, and 2) to support trainers who were not yet experts and thus still required our support (as do their staff, while the trainers were still being trained/supported to develop their skill and knowledge).

In mid-2016, the three-phase implementation program was introduced in recognition of the importance of organisational readiness in eMH implementation [14]. The program aimed to provide organisations with comprehensive support to create an optimal work environment for eMH adoption. Particularly, it focuses on providing organisational readiness consultations during pre- and post-training. The program was offered as an opt-in option for organisations in addition to the usual training and TtT workshops and follow-up support. The details of the program are described below.

### The 3-phase implementation program

#### Development of the e-index measurement tool

The 7-item e-Index measurement tool was developed as part of the implementation program. It is an organisational readiness measurement specific to eMH implementation aimed to be used as both a discussion and evaluation tool. The development of the tool is underpinned by the integrated Promoting Action on Research Implementation (*i*-PARIHS) theoretical framework [18]. The framework posited that successful implementation is achieved through a facilitation (**F**acilitation) process which assesses and aligns knowledge regarding the innovation to be implemented (**I**nnovation), the individuals and/or teams involved in the process (**R**ecipients), and the settings in which the innovation is to be implemented (**C**ontext). The e-Index items were further informed by review of other organisational readiness measures, e.g., the Organizational Readiness to Change Assessment (ORCA), and the Checklist to Assess Organizational Readiness (CARI) [19, 20], as well as themes from the qualitative data gathered during the first 3 years of the Menzies eMH training and implementation activities, and discussion within the research team.

The e-Index tool was used as part of the facilitation process to guide the organisation in evaluating levels of readiness and identifying improvements needed across the different aspects relating to eMH implementation. More specifically, the tool initiated discussions around research, clinical, patient and local knowledge of eMH (I), factors relating to the individuals and teams in supporting or resisting eMH approaches (R), and the nature and infrastructure of the organisation relating to eMH implementation (C). The tool was incorporated in the pre-training consultations and the follow-up phase within the implementation program.

##### Pre-training consultation

Prior to training, individuals involved with eMH implementation (the organisation’s internal facilitation team), e.g., the CEO, senior managers, general staff, and IT consultants from the organisation attended consultation workshop(s) where they completed the e-Index with an external facilitator (Menzies’ trainer). The external facilitator engaged participants in a group discussion around organisational readiness relating to eMH implementation. The e-index (described below) guided discussions across 7 key areas and enabled a consensus rating of organisational readiness for each aspect. Participants were encouraged to set goals and create an action plan to address areas identified as needing improvement. Internal trainers, who are responsible for conducting eMH trainings within the organisation, were also identified as part of the process. The aim of this phase was to prompt organisations to reflect, identify and set goals for change to render the work environment more conducive to eMH implementation, prior to receiving the training (i.e. prepare the context for maximum success).

##### Training

All participants received the same eMH training and TtT workshops, as described above.

##### Follow-up

In addition to the usual follow-up support offered to all organisations, the internal facilitation team from organisations enrolled in the implementation program attended follow-up face-to-face e-index consultation sessions (1 or 2 sessions) to evaluate progress and levels of organisational readiness, and to highlight need for further action.

For the purpose of this study, organisations enrolled in the implementation program were categorised as case studies, while those who did not opt in to receive this option were categorised as non-case studies. The key differences between case and non-case studies are twofold: 1) the case studies’ selected internal facilitation team received pre-training e-index consultation(s) to promote organisational readiness while non-case studies did not, and 2) in addition to the usual follow-up support offered to all organisations, case studies’ internal facilitation team also received additional e-index consultation(s) in order to evaluate and strengthen organisational readiness.

It is important to highlight that in 2016–2017, case studies received only one pre-training e-index consultation session and two post-training e-index consultation sessions. Upon reflection, it was recognised that organisations needed more support pre-training, and thus case studies in 2017–2019 received two pre-training e-index consultation sessions and one post-training e-index consultation session.

### Design

**Fig. 1:**
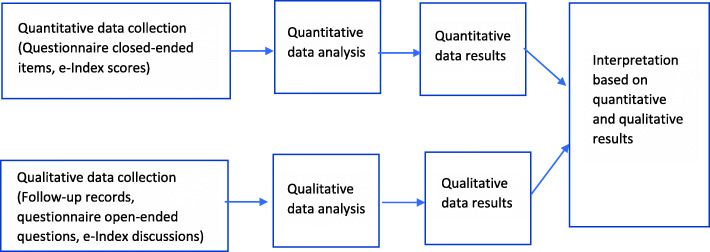
Triangulation design: complementarity model

A concurrent triangulation design, in accordance with Creswell and Plano Clark’s typologies of mixed methods design [21], was utilised to combine quantitative and qualitative data (Fig. 1). Quantitative data were collected through closed-ended items in the questionnaires and analysed using inferential and descriptive statistics. Qualitative data were collected via open-ended questions embedded in the questionnaires, consultation discussions, and follow-up support provided through emails, telephone calls and/or face-to-face sessions. Data were analysed using thematic analysis adapted from the steps described by Braun and Clarke [22].

The study’s design had complementarity functions which aimed to answer related questions for purposes of evaluation [23]. That is, quantitative methods were used to measure outcomes relating to the frequency of eMH usage and levels of organisational readiness, while qualitative methods were used to gain an in depth understanding of the implementation process. All of which were used to provide a comprehensive evaluation of the three-phase implementation program in enhancing eMH uptake.

### Participants and settings

From 2016 to 2018, 66 participants attended eMH training workshops which were held in remote Northern Territory communities, Darwin, Alice Springs, and Adelaide. Participants either self-selected or were selected by their organisations to attend the trainings. The workshops were advertised through professional networks, newsletters and the Menzies School of Health Research website.

All participants (from case and non-case studies) completed structured questionnaires incorporating visual analogue scales and open-ended questions prior to and post training. They were also offered on-going follow-up support for up to 12 months post-training, from which qualitative data were collected. The e-Index, however, was only completed by participants from case studies. Note that not all trainees accepted offers of follow-up support and remained engaged for the whole 12-month period. The main reasons cited for declining support were: no intention of using eMH approaches, movement to a different organisation, or changing to a different role that would not involve use of eMH. The demographic information of participants who consented for their data to be used for research purposes are reported in Table 1. Professions reported in ‘Other’ category included case manager, health promotion officer, program development and evaluation staff, dietician, and support worker. Non-government community included organisations such as alcohol and other drugs rehabilitation services, and family and carer support services. Ethics approval was granted by all relevant ethics committees (ref #HREC 12–1881, #CAHREC 12–100, and #AHREC 04–17-749).

**Table 1:** Participants’ profession and service type

Participants	N (%)
**Profession**	***N*** **= 65**
Indigenous Health Worker	3 (4.6)
Aboriginal Mental Health Worker	1 (1.5)
Aboriginal Community Worker	6 (9.2)
Alcohol and Other Drug Worker	4 (6.2)
Nurse	13 (20)
Psychologist	2 (3.1)
GP	1 (1.5)
Social Worker	9 (13.8)
Occupational Therapist	2 (3.1)
Trainer/Educator	3 (4.6)
Manager/Coordinator/CEO	6 (9.2)
Other	15 (23.1)
**Service Type**	***N*** **= 64**
Aboriginal Community Controlled Health Service	17 (26.6)
General Practice	1 (1.6)
Non-government Community	31 (48.4)
Government Community	8 (12.5)
Primary Health Care network	1 (1.6)
Other	6 (9.4)

#### Data collection

Organisational data were collected over 12 months. The final e-Index was completed 6 months post training workshop. Some quantitative and qualitative data were collected simultaneously, i.e., the post-training questionnaire and the e-Index included both closed and open-ended questions, and some were collected independently, i.e., records of follow-up support sessions collected qualitative data only, while the pre-training questionnaire included only closed-ended questions. Additionally, the e-Index was only completed by the case studies. What and how quantitative and qualitative data were collected is described in more detail below. The implementation matrix (Table 2) depicts a summary of data collection methods, whether it was collected from case or non-case studies, and what aspect of the research objective each data addressed.

**Table 2:** Implementation matrix

Research question	Data collection methods	Participants type
Do case studies evidence greater eMH usage than non-case studies?	eMH usage data reported in follow-up assessment	Case and non-case studies
	Number follow-up support records with evidence of eMH use	Case and non-case studies
Do case studies evidence improved organisational readiness as they progress through the implementation program?	e-Index scores	Case study only
How does the implementation program influence organisational readiness for eMH implementation?	Written records of follow-up support	Case and non-case studies
	Written answers to open-ended survey questions	Case and non-case studies
	Written records of discussions during e-Index completion consultation sessions	Case study only

#### Quantitative

##### The pre- and post-training evaluation questionnaire

The questionnaire included items assessing knowledge and confidence in eMH utilisation with a visual analogue of a 10-point Likert Scale. The questionnaires were adapted from the previous evaluation forms used in the ‘Yarning About Mental Health’ training in consultation with other eMHPrac partners. The questionnaire had been previously used to demonstrate effectiveness of the eMH training workshops [24].

##### E-index

The e-Index was developed by the research team as outlined above. It incorporated seven items assessing levels of readiness across various aspects within the organisation deemed essential to eMH implementation. These are: 1) Consultation - the extent to which consultation with experts, community and staff regarding eMH approaches has been carried out; 2) Resources – having appropriate and sufficient technological and human resources; 3) Systems fit – the extent to which eMH approaches fit with the existing systems and policies; 4) Implementation planning – extent to which the organisation has a clear plan for integrating eMH approaches as part of the organisation working culture; 5) Service provision fit – how well eMH approaches might integrate with staff’ day-to-day practice, 6) Integration through continuous quality improvement – extent to which the organisation has clear strategies for feedback and evaluation, 7) Change climate – taking major events and organisational changes into account, extent to which now is the best time for implementing eMH; 8) Additional question assessing participants’ levels of confidence in their ability to complete the e-index for the organisation.

Each item had two to five closed-ended (yes or no) questions with space for additional comments and justifications. At the end of each item, there was a visual analogue representing a 10-point Likert Scale which enabled an overall rating of the level of readiness, i.e., from ‘not at all’ to ‘fully ready’, for that particular item. The e-index was completed as part of the pre- and post-training consultation sessions which involved an external facilitator (a member of the research team) and representatives from the organisation, e.g., The CEO, general staff, senior managers, and IT consultants (the internal facilitation team). The external facilitator guided the e-Index completion by instigating a group discussion about e-Index items and recording relevant comments and the group’s consensus ratings for each of the item. The e-index is attached as a supplementary file.

##### Follow-up assessment

The follow-up assessment (completed 6-months post-training) included an open-ended question which assessed frequency of use across three Indigenous specific eMH tools: 1) Stay Strong app (SS) – a clinician-guided app using strengths-based approaches aiming to promote social and emotional wellbeing of Indigenous Australians; 2) Mindspot Indigenous Wellbeing Course – an online course designed to help Indigenous Australians manage symptoms of depression and anxiety [25]; 3) Stayin’ on Track – a website hosting resources aiming to support single Indigenous Australian fathers [26]. The number of uses is summed across all three eMH tools and is compared across case and non-case studies.

##### Follow-up support records

All participants were offered follow-up support in the form of emails, phone calls, and face-to-face consultation sessions. Phone calls and face-to-face discussions were recorded by the external facilitator in a written format immediately after the session. As part of the follow-up support discussions, participants were asked whether they had used eMH approaches. Therefore, participants whose follow-up record showed that they had used eMH approaches in their practice were counted separately for case and non-case studies. These numbers do not reflect the actual number of times these participants had used these approaches, but simply whether or not they had been used.

#### Qualitative

##### Follow-up support records

The follow up support record also documented participant’s ID, session type (e.g., phone call or group/individual face-to-face consultation), and participants’ reports on their experience of using the SS app and other eMH resources as well as any issues that hindered usage and integration of the approach into usual practice. Consultation sessions were not structured; rather they catered to participants’ specific needs. Therefore, records of the sessions reflected individuals’ and groups’ unique experiences in eMH implementation.

##### Open-ended questionnaire items

The open-ended questions embedded in the post-training evaluation and follow-up assessment sought participants’ experience of implementation challenges and their perception of organisational changes required to support eMH adoption.

##### E-index discussions

The e-index had space for comments related to each closed-ended question as well as to the justification of the overall score for each of the seven items. The written data is recorded by the external facilitator based on the group’s discussion. Additionally, an action list was created at the end of each of the consultation sessions detailing specific actions the organisation will take to improve its readiness for eMH implementation. The action list was first written down by the external facilitator on paper and was then translated into an electronic copy after the session. The electronic copy was then emailed to the participants involved in the session and a copy was kept for research purposes.

### Data analysis

Quantitative and qualitative data were analysed separately. T-tests and descriptive statistics were conducted to analyse quantitative data from the questionnaires using SPSS version 24.0, while the e-Index scores were entered and graphed in Microsoft Excel (2018).

Qualitative data were analysed using theoretical thematic analysis [22]. The analysis oscillated between deductive and inductive approaches. The data were first analysed inductively. After reading multiple times, common perspectives were grouped together with an assigned descriptive code name. Each code and its associated data extract were then reviewed and re-arranged into themes according to the *i*-PARIHS elements and sub-elements, i.e., innovation, recipients, context, and facilitation. Comparison revealed consistency across categories, and data saturation was reached.

First qualitative data from the e-Index discussions, i.e., written comments in the allocated space under each of the items in the e-index and the action list, was analysed following the steps described above. As the e-Index was completed as part of the implementation program, the qualitative data was only available for case study organisations. The qualitative data recorded as part of follow-up support, i.e., records of follow-up discussions via telephone, emails and group or individual face-to-face sessions, was analysed in a similar manner. This set of data was available across case and non-case studies as follow-up support was offered to all participants. The data from follow-up support was analysed in two ways. First, the data was pooled and analysed separately for each of the case studies. Then all of the data from the case studies were compared to those from the non-case studies.

The analytic process was conducted by the first author, BR. The final themes and sub-themes, their associated data extracts, and the interpretation of the data, were discussed within the research team to ensure rigour and validity.

## Results

### Quantitative findings

#### Training evaluation

Independent t-tests were conducted to assess whether case and non-case studies exhibited similar perception, knowledge and skills around eMH at pre- and post-training. Results showed that in general participants’ levels of confidence, skills and knowledge of eMH did not differ significantly across case and non-case studies. However, at pre-training, participants in the case studies *exhibited* higher confidence in communicating about wellbeing (*t* (60.218) = 4.068, *p* = .000) and perceived eMH to be more effective than those in the non-case studies (*t* (34.328) = 2.654, *p* = .012). While post-training, participants from the non-case studies exhibited higher levels of eMH knowledge than those in the case studies (*t* (64) = − 3.033, *p* = .003). The means and standard deviations are reported in Table 3. Note that 10 is the maximum score.

**Table 3:** Pre- and post-training ratings for case and non-case study participants

	Pre-training M (SD)		Post-training M (SD)	
Item	Case	Non-case	Case	Non-case
1. Confidence with wellbeing concerns	7.61 (1.16)	6.10 (1.80)	7.66 (1.09)	7.58 (1.47)
2. eMH knowledge	4.19 (2.16)	3.56 (1.85)	5.73 (2.13)	7.07 (1.45)
3. Ipad/tablet competency	7.26 (2.26)	6.96 (2.37)	7.63 (1.97)	8.00 (1.50)
4. Computer competency	7.51 (1.73)	7.75 (1.54)	7.91 (1.64)	8.33 (1.08)
5. Confidence in using SSA	5.88 (2.37)	4.85 (2.31)	7.31 (1.79)	7.67 (1.43)
6. Confidence in using eMH	5.84 (2.18)	5.37 (2.25)	6.94 (1.92)	7.35 (1.33)
7. eMH referrals competency	5.55 (2.41)	5.30 (2.31)	6.55 (2.10)	6.79 (1.53)
8. Accessibility to eMH	4.47 (1.73)	3.44 (1.93)	6.56 (2.00)	6.11 (1.93)
9. Appropriateness of eMH	6.29 (1.60)	5.73 (2.50)	7.42 (1.68)	7.10 (1.85)
10. Effectiveness of eMH	6.81 (1.11)	5.25 (2.49)	7.11 (1.81)	7.02 (1.73)

Paired *t*-tests were conducted to compare levels of confidence, knowledge and skills of eMH from pre- to post-training for all participants. Results showed significant improvements across all measures. Mean (M) and Standard Deviation (SD) are shown in Table 4. For the pre- training evaluation questions on ‘accessibility’, ‘appropriateness’ and ‘effectiveness’ of e-mental health for Indigenous people, 9, 11, and 19 participants respectively selected ‘don’t know’, indicating insufficient knowledge to answer the questions. However, fewer participants, i.e., 5, 5, and 7 participants selected ‘don’t know’ for the same questions post-training, indicating increased knowledge on the topics.

**Table 4:** Pre- and post-training ratings for all participants

Item	Pre-training M (SD)	Post-training M (SD)
1. Confidence with wellbeing concerns	6.76 (1.72)	7.63 (1.29) **
2. eMH knowledge	3.84 (2.00)	6.48 (1.89) **
3. Ipad/tablet competency	7.06 (2.32)	7.84 (1.72) **
4. Computer competency	7.67 (1.62)	8.15 (1.35) **
5. Confidence in using SS app	5.31 (2.38)	7.51 (1.61) **
6. Confidence in using eMH	5.58 (2.21)	7.14 (1.61) **
7. eMH referrals competency	5.41 (2.34)	6.68 (1.80) **
8. Accessibility to eMH	3.86 (1.96)	6.03 (1.94) **
9. Appropriateness of eMH	5.93 (2.23)	7.13 (1.76) **
10. Effectiveness of eMH	5.82 (2.21)	7.14 (1.78) **

*Note*. **p < .001

#### E-index scores

**Fig. 2:**
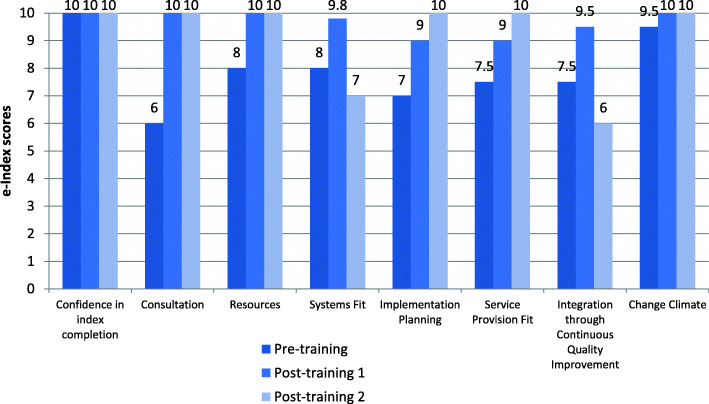
Organisation 1 e-index item scores

**Fig. 3:**
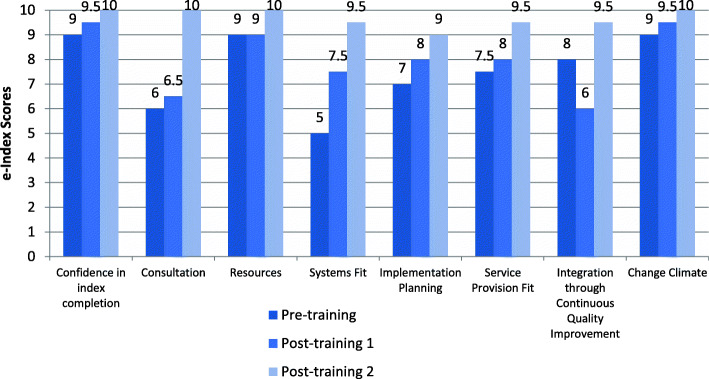
Organisation 2 e-index item scores

**Fig. 4:**
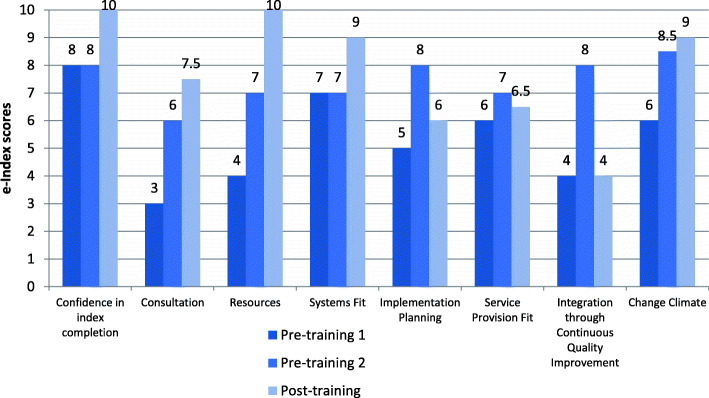
Organisation 3 e-index item scores

**Fig. 5:**
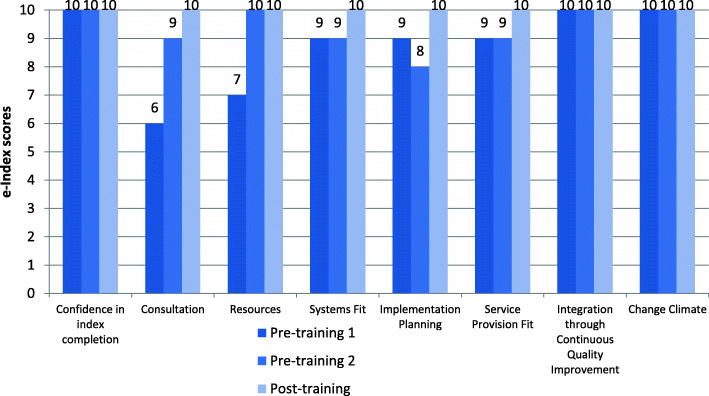
Organisation 4 e-index item scores

Organisations enrolled in the implementation program participated in three e-index completion sessions (averaging 1.5 h in length). Each session involved a combination of CEOs, senior managers, general staff, and IT consultants from the organisation completing the e-index, facilitated by a member of the research team. Participants from all four case studies were confident in their knowledge and experience to accurately complete the e-Index on behalf of their organisation. This is demonstrated by their ratings on the first e-Index item ‘confidence in index completion’ as 8 and above (Figs. 2, 3, 4 and 5).

In general, there was an upward trend on most of the e-index items for all organisations, with some exceptions: Organisation 1 scores for ‘system fit’ and ‘integration through continuous quality improvement’, and Organisation 3 scores for implementation planning’, ‘service provision fit’, and ‘integration through continuous quality improvement’ (see Figs. 2 and 4). Possible explanations for the lack of upward trend in some items are that the initial ratings were over-confident, that actions to accommodate e-MH were not achieved as predicted and/or that unforeseen implementation challenges arose. Nonetheless, the graphs depict improved organisational readiness across most aspects relating to eMH implementation for all organisations over the course of the implementation program.

#### eMH usage data

Follow-up assessment included a question regarding the frequency of use or referral to three culturally responsive eMH resources – the SS app, the Mindspot Indigenous Well-being course, and the Stayin’ on Track app. Due to a number of reasons including staff movement to a different organisation and disengagement from the research project, only 12 participants from the case studies and 3 participants from the non-case studies completed the follow-up assessment. In sum, participants from the case studies reported to have used or referred to eMH resources 67 times, while those in the non-case studies reported 7 times. Taking into account the difference in participant numbers, there is still a marked difference in use between the groups. i.e., on average participants in the case studies used eMH 5.58 times while those in the non-case used eMH 2.3 times.

Additionally, 6 out of 10 follow-up support records from the case studies demonstrated evidence of eMH use, in comparison to that of the non-case studies where only 2 out of 15 records exhibited evidence of eMH use.

### Qualitative analyses

These analyses seek to understand how the implementation program enhanced organisational readiness for eMH implementation.

The analysis is presented according to the *i*-PARIHS framework’s elements and sub-elements, i.e., innovation, recipients, context, and facilitation. Each of the elements and sub-elements include the following discussions:

- Barriers to eMH implementation for case and non-case studies
- A narration of how the facilitation strategies (i.e. e-Index discussion - for case studies, and follow-up support - for both case and non-case studies) helped to address the challenges.

It is important to highlight that due to a limited number of culturally responsive eMH approaches to Indigenous Australian populations, many of the participants reported to use the Stay Strong app which incorporates both Indigenous and Western perspectives of wellbeing [10]. Therefore, many of the responses reported here were related to the use of that app.

#### Innovation

Definition: knowledge of the innovation (information from research and clinical and patient experience) and its alignment with local priorities and practice.

##### *Underlying knowledge sources*

Participants from all four case studies reported to have limited awareness and knowledge of eMH approaches during the first e-Index completion session. Their knowledge, however, increased throughout the implementation program. They reported to have learned more about the approach through resources provided by Menzies and by seeking feedback from service providers’ and their clients’ experience using eMH.

##### *Degree of fit with existing practice and values*

During follow-up support, many participants from both case and non-case studies reported difficulties integrating eMH approaches into usual practice. There were two main challenges. One related to the use of the SS app with a group of clients: “*working with big groups of clients – difficult to give individual attention*”, and with couples: “*many staff are working with couples – this is presenting as an issue... A few staff were looking for solutions as the responses will be on one profile and the other person then feels it’s not theirs*.” Another difficulty identified was in the one to one setting where clinicians struggled to find time to have wellbeing conversations if their usual practice was focused on practical support.

“*Social support framework doesn’t allow the time to sit with people … too busy running around taking people to appointments … When people are focused on getting emergency services – housing, food, bills paid … there is no interest in doing the app*.”

Therefore, as part of follow-up support, Menzies staff helped participants to brainstorm ways in which eMH could be integrated into their usual practice. For instance, discussions confirmed that each client did not require a separate iPad - “*one iPad per support person rather than participant*”, or in a family setting that families could work together - “*short term solution to set up a 3rd profile which is a family one*”. Additional ideas to create opportunities to use the SS app such as “*offer food and sit with someone*” or “*sitting with people when on dialysis*” were also discussed.

##### *Usability*

The technical aspect of eMH approaches raised challenges for participants from both case and non-case studies. Participants reported difficulties with setting up the SS app (e.g., downloading the app, setting up passwords, registering emails etc. …), glitches within the app, and difficulty in printing care plan summaries. Additionally, having to remember to take iPads to appointments was a challenge for both groups. These issues were discussed, and solutions offered during follow-up support.

#### Recipients

Definition: Characteristics of the service providers (e.g., motivation, values, beliefs, skills and knowledge), and factors relating to the team’s culture, in supporting or resisting an innovation.

##### *Motivation & goal-setting*

The majority of the participants from both case and non-case studies demonstrated enthusiasm for using the SS app and eMH approaches and expressed the desire to use eMH more often in the future. However, records of follow-up support showed that only case study participants set explicit goals regarding when they would use the SS app and the number of clients with whom they would use it. Some case study participants also set plans to practice using the SS app and had discussions with researchers about using other eMH approaches.

##### *Skills & knowledge*

Levels of IT competency and knowledge of the SS app functions differed across participants. Some were more confident and competent using the app than others. Nonetheless, follow-up support provided participants from both case and non-case studies with solutions to technical issues, ‘how to’ explanations and revisions of the app’s functions (e.g., “*can go straight to Goal section, do not need to work through all app – menu bar on left hand side*”). For some participants from the case studies, follow-up support also provided discussions around counselling skills and how to best use the SS app in wellbeing conversations with clients.

“*Important discussion about not letting App drive the conversation, let the practice inform what goes into the app*.”

##### *Time, resources and support*

Limited time was a major impediment to eMH implementation for participants from both case and non-case studies. In follow-up support, participants reported difficulties finding time in their usual practice to incorporate the SS app.

“*All said they have so many resources and other things that they have to include, that it can be hard to add this in as well. None have had an opportunity to use it yet.”*

Participants from case studies also had difficulties finding time to discuss internal eMH training plans with their manager.

“*Difficulty in finding time to discuss training plans with manager. Unsure about expectations/training needs for team*.”

Lack of resources was an issue mainly for the participants in the non-case studies. The case study organisations made efforts throughout the implementation program to increase access and availability of iPads, tablets and WIFI. This was documented within the e-index discussions at which time the organisations made plans to install WIFI in certain areas and purchase additional iPad/tablets.

The implementation program also encouraged the organisations to develop a support system for staff around eMH use. The e-Index prompted the organisations to identify internal facilitators to undergo the TtT workshop, and to incorporate eMH discussions in individual supervision sessions and team meetings. Nonetheless, this was an on-going challenge for some organisations. For example, one organisation initially reported in the first e-Index completion: “*support in supervision sessions needs to happen. There has been minimal to no training in eMH”*, and while they made progress and reported having allocated time in supervision sessions to support eMH use, they still reported in the last e-Index discussion that staff needed more support. This was confirmed in the follow-up support discussions as participants from this organisation were not confident in using the SS app. They also reported having not received directions from management in terms of how to integrate the app into practice.

#### Context

Definition: different layers of context from the micro (local setting) through the meso (organisational setting) and macro levels (external health system) that act to enable or constrain implementation.

##### *Culture*

All four case studies reported a culture that is supportive of staff learning and development and described having allocated time and resources for on-going training of staff. During e-index completion, one organisation also discussed the idea of providing incentives for eMH use by giving acknowledgement through newsletters and for staff to include one case study in their quarterly reporting.

##### *Leadership and management support*

Support from management was identified by some participants in the non-case studies as essential to eMH implementation. This aspect was addressed in the implementation program which adopted a whole of organisation approach. Across all four case studies, the CEO, senior managers, IT consultants and other staff attended e-index completion sessions, demonstrating the organisation’s commitment to eMH implementation. Nonetheless, as discussed above, participants from the case studies reported during follow-up sessions that they felt the need for additional support to carry out internal eMH trainings and to integrate eMH into practice.

*“When asked if they had been given direction from management where/how to use the SS app, they hadn’t had direction but did know it was able to be inputted on database.”*

##### *Evaluation and feedback process*

Most of the case studies reported having an existing evaluation and feedback process prior to eMH implementation. For instance, one organisation described a continuous improvement system while another had client feedback integrated within their service closure process. Facilitated by the e-Index and follow-up support discussions, the organisations continuously adapted their system to incorporate feedback on eMH utilisation. Strategies such as “*put tablet/SS app usage as an agenda item for discussion at Team Meetings … and individual supervision sessions*” and “*have staff present to group on app use/experiences/thoughts on a rotating basis at team meetings*”, were brainstormed.

##### *Structure and systems*

High-staff turnover was common across case and non-case studies. A few participants who underwent eMH training left their organisation, and one internal trainer from a case study moved to another organisation.

Policy and protocols around IT use and data management were identified as a challenge to eMH adoption. In response to the open-ended question regarding changes needed in the organisation to implement eMH, the majority of the participants from non-case studies highlighted issues concerning policies and protocols:

“*Must be able to coincide with our policies*”

“*Systems around introducing iPads + downloading apps”*

The implementation program aimed to address this aspect by using the e-Index to instigate discussions around policies and protocols. This successfully encouraged the case studies to discuss and initiate plans to adapt, or create, policies and protocols for IT use and data management. By the end of the program, all four organisations reported progress in adapting or creating procedures around use of devices, data security and storage of information, and protocols for saving and transferring clients’ data. However, finalising and translating policies into practice was a slow process, as during follow-up support participants still reported: “*No consistent procedures around tablet storage, charging*”.

“*Staff aware that it is part of organisation policies and procedures and there is a movement towards it being used as an initial planning/assessment tool but still not being consistently used by all staff*”

Integration of eMH into usual practice was identified as a major challenge. Although the e-Index completion sessions prompted the organisations to develop a concrete plan for integration of eMH within their care-planning pathway, some participants still displayed uncertainty about how to incorporate the SS app in practice during follow-up support discussions.

“*Aware that managers are also discussing with Menzies changes to systems, processes etc. Unsure whether integrating app with assessment form will work – many assessments take place over the phone with people in remote communities*”

Follow-up support, therefore, provided another avenue to help participants brainstorm ways in which they could use eMH in their practice. For example, in a follow-up support session it was recorded:

“*Where does the SS app fit within practice framework was discussed. There was a lot of conversation around this as the staff had different ideas where they would use the tool within their practice … one is making plans for people, another is having the ipads in the drop in centre secured to ‘something’ and participants access the SS app on their own.”*

#### Internal facilitation

As part of the implementation program, the case studies identified an internal facilitation team. This team consisted of senior managers and IT consultants and included service providers who were responsible for conducting internal eMH trainings and providing eMH support and supervision to other staff (internal trainers). The internal trainers underwent the Train the Trainer workshop which was designed to provide them with skills and knowledge to carry out eMH training independently. Follow-up support discussions demonstrated evidence of internal eMH trainings being carried out in the case study organisations and the trainers’ plans for future trainings. During follow-up support, Menzies staff assisted the internal trainers with resources, presentation materials and space to discuss their training plans. Menzies staff also offered to come into the organisation and observe the trainers deliver their planned eMH training.

*“[participant] and [participant] stayed and developed a training for staff... Talked about catering and will provide afternoon tea. Developed a 2-3 hour SS App session delivered tomorrow*.”

## Discussion

The present study aimed to evaluate the effectiveness of the three-phase implementation program in enhancing e-mental health adoption within Indigenous primary healthcare organisations. Quantitative data were used to evaluate eMH usage outcomes and levels of organisational readiness, and qualitative data were used to gain insight into the process.

The 3-phase implementation program aimed to provide comprehensive support prior to and post eMH training workshops in order to help organisations build an optimal environment for eMH implementation. The present findings demonstrate that the organisations which opted into this program (case studies) exhibited greater use of eMH approaches than those that did not (non-case studies), suggesting effectiveness of the program in enhancing eMH uptake. It is important to highlight that both case and non-case studies similarly benefited from eMH training workshops, thus the difference in eMH utilisation between the two groups is not likely due to the differences in training. Moreover, follow-up support post-training was provided to all organisations for up to 12 months; therefore, the case studies’ greater use of eMH approaches was likely to result from the additional support provided as part of the implementation program, along with the likelihood that these self-selected organisations were possibly more committed to the adoption of eMH approach.

In addition to the training and follow-up support per usual, case study internal facilitation teams attended three consultations which focussed on organisational readiness. The e-Index was used in the consultation as a facilitation tool to guide the organisations in evaluating levels of readiness, and to identify and address areas that needed improvement to better support eMH adoption. Underpinned by the *i*-PARIHS framework [18], the e-Index specifically instigated discussions around knowledge of the eMH approach and its alignment with local priorities and practice, factors relating to the individuals and teams in influencing eMH uptake, and the organisational infrastructure essential to eMH implementation. In general, all four organisations demonstrated increased organisational readiness over the course of the program, as shown by the upward trend in the e-Index scores.

Taken together, the findings from case studies highlighted the importance of organisational readiness in supporting successful eMH implementation. This aligns with the existing literature which acknowledges the essential role of organisational readiness in successful implementation [27–31]. The average length of the implementation program was 12 months. The additional resource investment from the external facilitation team to the case study organisations was approximately 5 h per organisation.

To further understand how the implementation program enhanced organisational readiness, participant experiences from case and non-case studies were examined. Similar to the previous findings with Indigenous organisations, factors such as IT resources and infrastructure, leadership and support, policy and protocols around eMH utilisation and its integration into practice, were identified as essential to eMH uptake [8, 14, 15]. In accordance with the previous findings, issues such as insufficient technological and human resources, and not having clear policies and procedures around use of eMH approaches posed as barriers to eMH uptake. The program helped the case studies to work on and improve these aspects so that they better support eMH adoption. For example, the e-Index consultations prompted the organisations to audit their IT resources and they consequently purchased additional iPads, tablets, and improved WIFI connectivity. They also instigated the organisations to adapt, or develop, policies and protocols around use of devices, data security, storage of information, and transfer of clients’ data.

Moreover, given the important role of internal facilitators in successful implementation [32], the program also encouraged the organisations to select internal trainers who were responsible for conducting eMH trainings and providing implementation support and supervision to other staff within the organisation. The trainers underwent the TtT workshops, as part of the program, and received additional support post-training, e.g., follow-up discussions around internal training plans and training materials. Lastly, and arguably the most important point of distinction, was that case studies incorporated a whole of organisation approach where the CEO, senior managers, IT consultants, internal trainers, and general staff were included in the implementation process. This aligns with the organisational literature which deems commitment to change by the whole organisation as an essential precursor to successful implementation [33–35].

Nonetheless, although the e-Index prompted the case studies to identify where and how the eMH approaches would fit in with the existing care-planning pathway, integration of eMH into usual practice was identified as an on-going challenge. Issues such as limited time due to high workload and competing priorities (i.e., practical needs of clients for support with housing, food or finance), and practice frameworks that did not allow for one-on-one time, were highlighted as significant barriers. Previous findings with Indigenous organisations also found similar issues [14, 15]. Follow-up support attempted to address these challenges by helping the service providers brainstorm ways in which they could adapt their usual practice to include eMH approaches.

Another important aspect is the appropriateness of the eMH resources for Indigenous clients. Previous research demonstrated the importance of eMH resources to be culturally responsive to Indigenous Australians. This notion was expressed by both community members and service providers [8, 9]. Therefore, knowledge and selection of culturally appropriate eMH resources is an important part of the implementation process. This was incorporated in the implementation strategies in a number of ways. In the training workshops and implementation support, one main focus was providing service providers with the knowledge and skills to select and use credible eMH resources that have been developed with and for Aboriginal and Torres Strait Islander people, e.g., the Stay Strong app. Additionally, as part of the e-index discussions, a number of items encourage direct involvement of clients when deciding to use a eMH resource.

Taken together, these findings suggest that primary healthcare services working in Indigenous context may benefit from a more comprehensive implementation program such as this one, which focuses on selecting culturally appropriate eMH resources and provides extensive support, from implementation strategies for the day-to-day practice to organisational infrastructure and systems. As demonstrated by the present study, a whole of organisational approach with extensive consultations can help such organisations address key issues and barriers and build capacity for a successful implementation of eMH approaches.

The present findings benefit Indigenous primary care consumers in a number of ways. Firstly, they contribute to the growing knowledge and understanding of the implementation process of eMH approaches in organisations that work with Indigenous clients. Secondly, they offer effective implementation strategies, which enable service providers to appropriately use eMH resources and realise the approach’s benefits for the community members, particularly for those who live in remote areas. Lastly, given our position in the eMHPrac project (a government-funded project which supports the utilisation of eMH approaches), the findings can be used to advise the government on how implementation could be better supported in organisations providing services to Indigenous consumers.

It is important to highlight that Cultural Consultants were heavily involved in the research process, from resource development to evaluation. Whenever possible, at least one Cultural Consultant helped facilitate the training workshops and e-index discussions. Their feedback during debrief at the end of the sessions were insightful and helped shape our understanding and evaluation of the activities. The organisations themselves were also encouraged to nominate at least one cultural advisor to attend the e-index discussions.

The e-Index consultations can be considered as a co-evaluation. At each session, service providers and the Menzies facilitator work together to evaluate the organisation’s progress, i.e., identifying remaining key issues and developing plans going forward. Therefore, progress evaluation and the implementation plans are specifically tailored to the individual organisation. At the end of each session, a summary and action list is sent to the organisation to review. Additionally, some participants who attended the Train the Trainer workshops were Indigenous service providers who were upskilled and went to play roles in their organisations as trainers, facilitating eMH training workshops internally. They also attended the e-index discussions and provided insightful perspectives to the evaluation from that of an Indigenous person, a service provider, and an internal facilitator. Therefore, the implementation program was undertaken through extensive collaborations between Menzies and Indigenous organisations, as well as other organisations that provide services to Indigenous clients.

Another important issue to note is that the e-Index, though a useful discussion tool, is not an optimal evaluation tool. High scores on the e-Index for certain aspects often did not accurately reflect on the ground practice. For example, Organisation 2 scored quite highly on ‘implementation planning’ and ‘service provision fit’; however, participants from the organisation reported need for more support and direction from management regarding the fit of eMH within the care-planning pathway and day-to-day practice. This pattern was similarly reflected in Organisation 1. Additionally, although all case studies reported to be fully resource ready, participants from some organisations still identified access to IT resources as an issue.

Given the advantage of having e-Index guided organisational readiness discussions and yet the shortcomings of the tool in accurate readiness assessment, it would be fruitful for future studies to develop a valid and reliable measure of organisational readiness specific to eMH implementation. The measure could function as a diagnostic tool. That is, it could be used to assess the initial level of organisational readiness to help determine optimal commencement time for eMH implementation to maximize uptake. The tool should focus on specific and concrete aspects of organisational readiness, while minimising potential for scores based on opinions and unfounded optimism. Additionally, it could also be used throughout the implementation process to track progress in organisational readiness and to help identify areas that need further improvement. Importantly, a valid and reliable measure would enable future studies to conduct rigorous quantitative analyses and draw inferential conclusions regarding the impact of organisational readiness on eMH implementation outcomes.

The present study is not without limitations. Due to several reasons, as noted in the methods section, a small number of participants completed the follow-up assessment and participated in follow-up support. Therefore, data on the use of eMH approaches may not accurately represent, and may underestimate, actual use within the organisation. Additionally, experiences reported in the follow-up support records may also not fully represent the experience of others in the organisation. However, the themes identified here are similar to those of previous studies within the field [8, 14, 15]; thus, provide a level of confidence regarding the validity and reliability of the present findings. Another limitation is that the consumers’ voices were not heard separately to that of the service providers’ during the e-index consultations and follow up interviews. Their preferences and experiences were relayed indirectly through the service providers during the discussions. However, it is important to note that Indigenous community members and Indigenous service providers collaborated in the development and evaluation of the Stay Strong Plan which preceded transition to the app [36] and in the transition of the paper based plan to the Stay Strong App. Furthermore, cultural advisors and Indigenous co-trainers were involved in the training workshops and e-index discussions whenever possible.

In addition, case studies were self-selected and thus it is likely that these organisations commenced the process with greater willingness to adapt their systems and processes for eMH and invest in implementation. Nevertheless, the combined data reveals engagement with the implementation support and responsiveness to the guidance offered. Lastly, most of the qualitative data, i.e., follow-up support records and e-Index discussions, were recorded by research team members. Thus, the reported experience may have been subjected to perceptual bias on the part of the recorder. However, measures were taken to reduce bias as much as possible, i.e., records of e-Index discussions and follow-up support were taken during or straight after the discussion, and the e-Index and the action lists were sent to the organisation for their perusal.

## Conclusion

Taken together, the present findings demonstrated the effectiveness of the three-phase implementation program in enhancing eMH uptake within Indigenous primary healthcare organisations. The program empowered organisations to engage, evaluate and take actions to improve on multiple aspects essential to eMH implementation. Therefore, by enhancing organisational readiness, the program helped the organisations to better support eMH implementation and thus enhanced uptake.

## Data Availability

The datasets generated and/or analysed during the current study are not publicly available due to privacy concerns but are available from the corresponding author on reasonable request.

## Abbreviations

eMH: e-mental health
eMHPrac: e-Mental Health training and support project
*i*-PARIHS: The integrated Promoting Action on Research Implementation framework
IT: Information Technology
Menzies: Menzies of School of Health Research
TtT: The Train the Trainer workshop
